# Screening test for neutralizing antibodies against yellow fever virus, based on a flavivirus pseudotype

**DOI:** 10.1371/journal.pone.0177882

**Published:** 2017-05-31

**Authors:** Séverine Mercier-Delarue, Christine Durier, Nathalie Colin de Verdière, Jean-Dominique Poveda, Vincent Meiffrédy, Maria Dolores Fernandez Garcia, Stéphane Lastère, Raymond Césaire, Jean-Claude Manuggera, Jean-Michel Molina, Ali Amara, François Simon

**Affiliations:** 1 Department of Microbiology, Saint Louis University Hospital, Paris, France; 2 INSERM—SC10-US19,Villejuif, France; 3 Department of Infectious Diseases, Saint Louis University Hospital, Paris, France; 4 Laboratoire CERBA Saint-Ouen-L'aumône- France, Saint-Ouen-L'aumône, France; 5 INSERM U944 -UMR 7212, Saint Louis University Hospital, Paris, France; 6 Department of Medical Biology, Centre Hospitalier de Polynésie Française, Papeete–Tahiti, French Polynesia; 7 Department of Viro-Immunology, University Hospital of Fort de France, Fort de France- Martinique, French West Indies; 8 Institut Pasteur, Environment and Infectious Risks Research and Expertise Unit, Laboratory for Urgent Response to Biological Threats, Paris, France; University of Pittsburgh Centre for Vaccine Research, UNITED STATES

## Abstract

Given the possibility of yellow fever virus reintroduction in epidemiologically receptive geographic areas, the risk of vaccine supply disruption is a serious issue. New strategies to reduce the doses of injected vaccines should be evaluated very carefully in terms of immunogenicity. The plaque reduction test for the determination of neutralizing antibodies (PRNT) is particularly time-consuming and requires the use of a confinement laboratory. We have developed a new test based on the use of a non-infectious pseudovirus (WN/YF17D). The presence of a reporter gene allows sensitive determination of neutralizing antibodies by flow cytometry. This WN/YF17D test was as sensitive as PRNT for the follow-up of yellow fever vaccinees. Both tests lacked specificity with sera from patients hospitalized for acute Dengue virus infection. Conversely, both assays were strictly negative in adults never exposed to flavivirus infection or vaccination, and in patients sampled some time after acute Dengue infection. This WN/YF17D test will be particularly useful for large epidemiological studies and for screening for neutralizing antibodies against yellow fever virus.

## Introduction

Yellow fever virus is an extremely dangerous pathogen transmitted by *Aedes* and *Haemagogus* mosquitoes. Recent literature reviews highlight a risk of transmission of this virus to currently preserved areas and the need to confront future large epidemics, a scenario that could be complicated by a lack of vaccine [1,2]. Since its development in the 1930s, the live attenuated vaccine against yellow fever (AAV) has been widely used, and an estimated 60 million doses are administered each year [3]. This vaccine confers protection in almost 100% of cases, and neutralizing antibodies can be detected in more than 90% of patients 10 days after vaccination and in more than 99% of patients after one month [2].

Until now, the protection given by the yellow fever vaccine has been estimated to last about ten years, but several studies suggest that this immunity could last for up to 30–35 years or even lifetime. The World Health Organization (WHO) has issued a report on yellow fever vaccination, concluding that the immunity given by this vaccine is stable over time and that a single dose of yellow fever vaccine is probably sufficient, with few exceptions, to provide lifetime immunity [4,5]. Consequently, at least in adults, there would be no need to re-administer a booster dose after 10 years, thus avoiding the risk of rare but serious post-vaccination complications and also sparing vaccine doses [6,7]. Indeed, worldwide production of yellow fever vaccine is relatively limited, with only 5 manufacturers producing 100 million doses per year. Despite the creation of an emergency stockpile by WHO, the lack of vaccine is a major risk to global public health. A reduction in the injected volume has been proposed to increase the number of vaccinations during mass campaigns, thus limiting the risk of side effects and reducing public health expenditure. Such a reduction in the injected dose, as well as combined administration with other vaccines or using other routes of injection, such as the intradermal route, must be closely evaluated in terms of immunogenicity, along with new vaccines such as the inactivated yellow fever vaccine [8–10].

The main visceral complication of vaccination is Vaccine-Associated Viscerotropic Adverse Events, particularly at the time of primary vaccination, with a high mortality rate. Post-vaccine neurological disorders may also occur after booster doses, suggesting an autoimmune phenomenon. The pathophysiology of these accidents is not fully understood and the most clearly identified risk factors are age over 60 years, a history of thymic disorders or thymectomy, as well as autoimmune diseases or genetic defects of innate immunity **[6]**.

Finally, the increasing number of immunosuppressed travellers (patients living with HIV, cancer or immunosuppressive treatments) requires specific monitoring of the risk-benefit ratio of yellow fever vaccination. Evaluation of vaccine protection in immunocompromised populations, as well as new dose reduction strategies, can only be evaluated in terms of immunogenicity by using surrogate biological markers, neutralizing antibodies being considered the gold standard [11]. The detection of neutralizing antibody activity is based on the reduction in the number of plaques formed by the amaril virus in cell culture by antibody neutralization (PRNT). This laboratory test, developed some 50 years ago, can only be done in specialized laboratories [12]. The PRNT has several other limitations which were recently reviewed by Jean Jonkert *et al* [13]. The PRNT measures the ability of a serum sample to neutralize yellow fever virus in cell culture, with a constant amount of serum and a variable amount of virus or, more often at present, a variable amount of serum and a constant dose of virus. The most widely used PRNT is based on a standardized amount of YFV17D yellow fever virus capable of forming 50 to 100 plaques in cell culture, to which serum is added at several dilutions. The correspondence between protection against yellow fever virus and the PRNT dilution titre was established in monkeys infected with yellow fever virus [14]. Survival was good when the sera were capable of reducing plaque formation by at least 80%. The detection limit in humans is considered to correspond to 50–80% plaque reduction with 1:10-diluted serum. This test is labour-intensive, and only a limited number of samples can be tested in each run. Several adaptations have been proposed, including a micro test to reduce inter-test variability, but the biggest limitation of PRNT is the need for 5 to 6 days of culture, and for highly experienced technicians to read the plaque inhibition [15,16]. Finally, the viral strain must be manipulated in a safety laboratory. However, inhibition of virus growth in culture remains the best means of evaluating the immunity induced by a live vaccine. A simpler, faster method capable of testing a large number of samples simultaneously is obviously desirable. The NCT014 26243 study, referred to below as NOVAA (French acronym for "new tools for yellow fever vaccination"), is an interventional study sponsored by ANRS and designed to assess the immune response and virological markers after vaccination of naïve adults. The study is a 5-year follow-up of the response to the 17D-204 vaccine (Stamaril®, Sanofi Pasteur, France). As an ancillary study to this protocol, we developed and evaluated a new test based on the use of a pseudoviral vector combining the genes of West Nile and YFV17D virus releasing pseudotype virus-like particles (VLP) for simple, quantitative and repeatable measurement of neutralizing activity following 17D-204 vaccination.

## Materials and methods

### Serial sera from 17D-204 vaccinees

To calibrate the NOVAA test, we first compared the results with those of a panel of 20 stored sera previously tested with our PRNT assay. Then, we used both assays to test samples prospectively collected by phlebotomy on day 0 (D0) and at one year (M12) in the NCT01426243-ANRS study. Thirty healthy adults resident in France, who had never been vaccinated against yellow fever and who had never stayed in an endemic country were included. The subjects were seronegative for HIV, HBV and HCV, and women had a negative pregnancy test. The subjects were vaccinated with STAMARIL® 0.5 ml subcutaneously, according to the manufacturer's recommendations. 17D-204 STAMARIL® E5499-1, extensively characterized elsewhere, was the only batch used in this study [17]. Day 0 sera were used to determine specificity and reproducibility. M12 sera were used to establish sensitivity and reproducibility.

### Plaque reduction neutralizing reference test (PRNT)

Samples were assayed twice in parallel in two-fold dilutions ranging from 1:10 to 1:80 (final) in the plaque reduction neutralization test. The neutralizing antibody titer was recorded as the reciprocal of the highest serum dilution reducing by at least 80% the number of cell clusters infected by 100 PFU/0.1 ml YFV virus. Sera were inactivated at 56°C for 30 min and diluted 1:5 in Leibovitz L15 culture medium. Serial two-fold dilutions were then prepared from the 1:5 dilution. An equal volume of YFV-17D vaccine, calculated to yield approximately 100 PFU/0.1 ml, was added to each serum dilution and held overnight at 4^°^C. The next day, the mixtures were inoculated in duplicate in 24-well plates. PS cells (10^6^ cells/ml) were added to each well and the plates were placed in a 37°C incubator for approximately 4 h (ref 18). Inoculated wells with confluent PS cell monolayers were then overlaid with carboxymethylcellulose (CMC) diluted 1:2 in L15 medium and placed at 37°C for 5 days. The CMC medium was then discarded and the cell monolayers were stained with blue-black. The neutralizing antibody titer was expressed as the reciprocal of the highest initial serum dilution inhibiting at least 80% of plaque formation compared with the virus control titration. YFV antibody titers ≥ 1:10 were used as a surrogate marker of clinical protection.

### Pseudotype-based NOVAA-test

The *in vitro* NOVAA-test is based on cell culture with non-infectious pseudotype virus-like particles (VLP). A West Nile Virus—YFV17D pseudotype virus (WNV/YFV17D-VLP) allows a single round of cell infection, which is quantified in Vero cell culture by cytometry with a green fluorescent protein (GFP) reporter [18]. Yellow fever neutralizing antibody titers are expressed as the inhibitory activity of human serum against WNV/YFV17D-VLP.

#### Cell culture

HEK 293T/17 cells (ATCC^®^ CRL-11268™) were maintained in Dulbecco’s modified Eagle's medium (DMEM, Gibco^®^ Life Technologies™) supplemented with 10% heat-inactivated fetal bovine serum (FBS), 1% penicillin/streptomycin (P/S) and 1% L-glutamine in a 5% CO_2_ incubator at 37°C. For transfection, HEK 293T/17 cells were maintained in DMEM Glutamax supplemented with 10% FBS, 1% L-glutamine and 1% HEPES.

Vero cells (ATCC^®^ CCL-81™; http://www.lgcstandards-atcc.org/en.aspx) were maintained in 10% DMEM. For the NOVAA-test, Vero cells at 50% confluence were supplemented with 2% heat-inactivated FBS, 1% penicillin/streptomycin (P/S), 1% L-glutamine and 1% HEPES.

HEK 293T/17 transfections and Vero cells infections are performed when the cells in culture reached 50% confluence.

#### Plasmid viruses

Plasmid pACNR-FLYFV17DII expresses the full-length infectious 17D genome (a gift from P. Bredenbeek, Leiden University, The Netherlands). YFV 17D C- PrM-E genes were amplified from pACNR-FLYFV17DII and cloned as BamHI- and XhoI-digested fragments into a likewise pcDNA3.1-digested plasmid (Invitrogen, Thermo Fisher Scientific, Waltham, MA) [19].

The second plasmid WNVVII reporter–GFP, containing the NS1-NS2-NS3-NS4-NS5 region from the lineage II strain of West Nile Virus (strain WNV 956 D117 3B) encoding a subgenomic GFP replicon was provided by T. C. Pierson (NIH, Bethesda, MD) [20].

#### Transfection for WNVV/YFV17- VLP production

Reporter viral particles were produced by cotransfection of HEK 293T/17 cells with WNVVII-GFP and the PcDNA3.1 YFVV 17D plasmid. HEK 293T/17 cells in 6-well culture plates at 450 000 cells/well in 2 ml of DMEM Glutamax/10% FBS on day 0 were transfected using the CaCl2 method on Day 1.

Tube A contained per well: PcDNA3.1 YFVV 17D plasmid 3µg, WNVVII-GFP 1µg, 10µL and TE (Tris-EDTA) 0.1X in a final volume of 100µL.

Tube B contained per well: HPB 2X 100µL (5M NaCl, 10ml 0.5M HEPES pH7.1, 0.15M Na2HPO4.7H2O). Drop by drop, tube A was mixed with tube B, and the solution was overlaid on HEK 293T/17 6-well culture plates containing 2 ml of DMEM Glutamax after 20 min at room temperature. Two days after transfection, the supernatant containing VLPs was collected on ice, filtered (0.44 µm pore size) and stored at -80°C.

#### WNV/YFV17- VLP quantification

On Day 0, Vero cells were adjusted to 16 000 cells per well in 48-well plates with 500µL DMEM/10% FBS. On Day 1, VLP were diluted 1:2, 1:4 and 1:8 in 250µL of DMEM 2% FBS and incubated for one hour at 37°C. After discarding the medium, 250µL of the VLP dilution was added per well and incubated for one hour at 37°C. The volume was then adjusted to 500µl with DMEM 2% FBS and incubated for 48 hours at 37°C.

After two days, the medium was discarded and the cells were treated with trypsin (300µL) and collected with 700µL DMEM 10% FBS. After centrifugation at 1300 rpm for 3 minutes, the cell pellet was washed by centrifugation with 700µL of PBS and resuspended in 200µL of PBS in Micronic tubes (http://www.dutscher.com). The percentage of infected cells was quantified by flow cytometry (FACS Calibur, Becton Dickinson, San Jose, CA).

In order to compensate for the biological variability of cell culture and to observe a clear drop in the presence of neutralizing antibodies, the VLP working dilution must infect 20–30% of Vero cells.

#### The NOVAA-test

On Day 0, Vero cells were adjusted to 16 000 cells/well in 500µL DMEM 10% FBS in 48-well plates. The test serum (6µL) was diluted 1:10 with 54 µL in a negative control serum collected from a yellow fever-negative donor not having visited countries endemic for flaviviruses. 120 µL of VLP working dilution was added to 60µL of diluted serum and DMEM 2% FBS was added to a volume final of 600 µL. A control with a negative serum, VLPs and DMEM 2% FBS was systematically added. The serum and VLP were incubated at 37°C for one hour and 250µL was distributed in duplicate in 48-well plates without medium (https://www.fishersci.com) and incubated for one hour at 37°C. DMEM 2% (250µL) was added and the plates were incubated for 48 hours at 37°C. The cell monolayer was collected as described for WNVV/YFV17- VLP quantification.

#### Flow cytometry and expression of results

WNVV/YFV17D- VLP infectivity was expressed as the percentage of Vero cells expressing GFP activity in negative controls (%NC), considered to represent 0% neutralizing activity. Calibration used 5x10^3^ negative control cells. The percentage of infected cells in the wells with human sera is to represent neutralizing activity (% NA). Cells collected from the different wells were analyzed with the same procedure. Results were expressed as 1–(%NA/%NC) x 100, 100% representing 100% neutralization by yellow fever antibodies. Data were analyzed with Cell-Quest and Flow-JO software (BD Sciences).

#### Reproducibility of cell infectivity between different VLP productions

Intra- and inter-assay variability was determined by testing five negative sera at final dilutions of 1:100 in duplicate and in three runs on the same day with the 4 VLPs so far produced. The same procedure was performed the following day in order to study inter-assay variability.

#### Evaluation of NOVAA-test sensitivity for neutralizing antibody detection

Twenty stored sera that had previously tested positive in the PRNT were studied with the NOVAA-test to compare the two assays and to establish the positivity threshold for the NOVAA-test. Complementarily, due to a limited number of sera exhibiting 1:10 antibody titer by PRNT considered as a clinical protection, four sera exhibiting a 1:40 antibody titer by PRNT were diluted at 1:4 dilution and tested in NOVAA-test.

Using the sera from the 30 subjects collected prospectively before and one year after YFV-17D vaccination in the NCT01426243-ANRS clinical study, the PRNT and NOVAA-test were run in parallel. The lower limit of detection in the NOVAA-test was determined according to PRNT positivity.

A further sensitivity study was performed with both tests, using 1:100; 1:500 and 1:1000 diluted sera.

#### Evaluation of NOVAA and PRNT specificity

Specificity was determined on panels tested with both the NOVAA and PRNT assays. The first panel corresponded to the 30 Paris residents naive of vaccination and having never traveled to endemic areas at their inclusion in the study. The second panel was from 18 AAV-naive hospitalized children/young adults, collected during acute Dengue virus infection in the West French Indies, a region free of yellow fever and Zika virus at time of sampling. Dengue was confirmed by the presence of IgM and/or IgG (Immuno Essai Luminex 9-plex, Institut Pasteur, France). The third panel corresponded to sera collected from 33 unvaccinated young Polynesian adults living in Papeete, Tahiti, French Polynesia, a country endemic for Dengue and Zika viruses. These 33 patients were hospitalized for clinical Dengue, confirmed by the presence of IgM and/or IgG in EIA tests (SD Dengue IgG or Ig M Capture ELISA, Standard Diagnostics, Alere Waltham, Ma). The fourth panel corresponded to 23 healthy Polynesian blood donors with anti-Dengue IgG and a history of biologically confirmed Dengue. These Polynesian blood donors' sera were all negative by Dengue RT-PCR and negative for anti-Dengue IgM.

### Statistical methods

An ISO 5725–2 standard method was used to determine the repeatability (intra-assay precision) and reproducibility (inter-assay precision) of the percentage of infected cells [21]. Each serum was tested during two days in 3 runs. The reproducibility variance was the sum of repeatability and between-run and between-day variances. The different variabilities were estimated in a common model combining infectivity values of the five sera, using SAS 9.3 software. The results were expressed as percentage coefficients of variation (CV).

### Ethical considerations

All the subjects signed an informed consent form registered with the French National Agency for Drug Safety (ANSM) A100972-82, and ethical approval (10057) was delivered by the Ile de France 3 Ethics Committee. Sera were used in accordance with French Public Health Law Art. L 1121–1.1.

## Results

### VLP cell infectivity

Different WNV / YFV17D pseudoviruses were produced and the VLP products were designated VLP 2011, VLP 2013, VLP 2014 and VLP 2015. All the results presented here were obtained with VLP 2013. VLP 2013 infected approximately 20% of Vero cells, a percentage considered ideal for clear evaluation of cell infectivity and neutralization in the NOVAA-test (Table 1).

**Table 1:** Inter and intra-assay variability of infection with WNVV/YFV17D VLP 2011, VLP 2013, VLP 2014, VLP 2015 in Vero cell lines in 5 different sera collected before 17D- vaccine.

VLP	VLP 2011	VLP 2013	VLP 2014	VLP 2015
**Dilution**	1/100	1/100	1/100	1/100
**Mean % infected cells**	7.6	24.8	7.2	42.4
**s**_**R**_	2.3	6.4	0.9	10.2
**s**_**r**_	1.1	2.8	0.6	3.8
**%CV**_**R**_	30.3	25.8	13.2	24.1
**%CV**_**r**_	15.0	11.3	7.7	9.0

s_R_, %CV_R_: reproducibility (inter-assay) standard deviation and % coefficient of variation

s_r_, %CV_r_: repeatability (intra-assay) standard deviation and % coefficient of variation.

Preliminary results after one year of storage at -80°C showed that this infectivity of VLP 2013 fell from 25% to 15% (data not shown). This underscores the need to properly calibrate the VLP inoculum before use, in order to maintain a 20% percentage of infected cells before the NOVAA-test.

### Reproducibility of infection and variability of VLP infectivity

The results for the 5 negative sera collected before vaccination were compared at final dilution of 1:100. Table 1 summarizes the inter-variability and intra-variability of cell-infectivity, along with the mean and standard deviation of the coefficients of variation (CV) for each VLP and different serum dilutions. The repeatability CV was 7.7% to 15.0%. The inter-variability between tests done on different days ranged from 13% to 30%. The repeatability was below 20% of the CVs, showing satisfactory level.

### Sensitivity of NOVAA-test versus PRNT and detection limit for neutralizing antibodies

**Fig 1:**
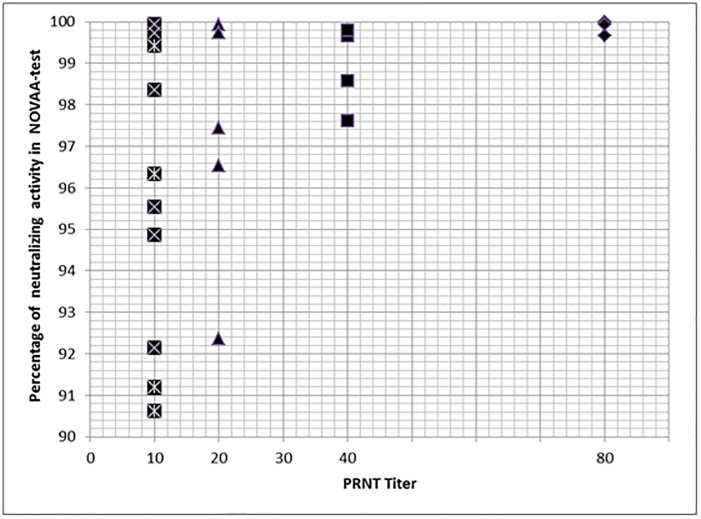
NOVAA-test results: Percentage of neutralization with 20 sera from patients with PRNT titres ranging from 10 to 80. Four complementary sera with a previous PRNT results of 40 were diluted 1:4 in order to surround the cut-off for neutralizing antibody detection by PRNT.

Twenty sera with known PRNT titres ranging from 10 to 80 and four diluted sera were first used to calibrate the NOVAA-test. These sera yielded more than 90% neutralization in the NOVAA-test regardless of the PRNT titre (Fig 1). Similarly, the samples collected at M12 following vaccination in the NCT014 26243 study showed greater than 90% neutralization in the NOVAA-test. The PRNT neutralization titres ranged from 40 to 80.

A further study was performed with 5 of these high-dilution sera at 1: 100, 1: 500 and 1: 1000 (Table 2). The NOVAA and PRNT tests both remained positive at the 1: 500 dilutions and gave a similar rate of positivity at 1: 1000 dilution. All together, these results suggested that the NOVAA-test detected active neutralizing antibodies when neutralizing activity reached at least 90%. Between 75% and 89%, the results should be considered to lie in a gray area pending further studies. Owing to potential biological variability, percentages below 75% should be considered insignificant.

**Table 2:** Neutralizing activity in the NOVAA-test and PRNT titers at 1:100. 1:500 and 1:1000 dilutions.

	NOVAA-test Dilution			PRNT -Dilution		
Sera ID	1/100	1/500	1/1000	1/100	1/500	1/1000
63002	100	100	100	80	20	<10
63010	100	97,66	72,85	40	10	<10
63012	100	100	97,74	40	10	<10
182003	100	99,37	71,58	80	10	10
73002	100	93,93	58,57	80	10	10

Positivity criteria: 90–100% (bolded numbers) neutralizing activity in the NOVAA-test is considered significant; 75–89% is considered indeterminate; and below 75% is considered uninterpretable. PRNT is considered positive when the titer is at least 10 (bolded numbers).

### NOVAA-test specificity

The 30 adults living in Paris without any exposure to flaviviruses were all negative in both the NOVAA and PRNT tests. Among the 18 Caribbean patients with acute Dengue, only 13 were strictly negative (44%) or in the gray area (N = 7, 38%) in the NOVAA-test, while 8 (44%) were strictly negative in the PRNT test (Table 3).

**Table 3:** NOVAA-test and PRNT values for 18 stored sera collected during acute clinical Dengue in hospitalized children, West French Indies.

ID	Age (y)	IgM	IgG	Neutralization NOVAA-Test %	PRNT titer
**8113**	3.6	positive	negative	39.14	<10
**7070**	18.5	positive	positive	37.71	<10
**22100**	1.8	positive	negative	26.29	<10
**71**	0.9	positive	negative	32.57	<10
**4053**	18.3	positive	positive	73	**20**
**30164**	16.6	positive	positive	75.09	**20**
**15008**	17.7	positive	positive	80.89	**10**
**24**	7.8	positive	positive	81.23	**20**
**2067**	0.9	positive	negative	84.66	<10
**22051**	18.5	positive	positive	79	**10**
**10135**	8.7	positive	positive	**92.37**	**20**
**3173**	6.9	positive	positive	89.71	**40**
**7094**	8.5	positive	positive	**97.14**	<10
**3031**	18.8	positive	positive	**100**	**80**
**25052**	13.5	positive	positive	**100**	**40**
**2061**	15.8	positive	positive	**99.89**	**80**
**12002**	17.6	negative	positive	57.83	<10
**11003**	3.1	negative	positive	81.71	<10

Positivity criteria: 90–100% (bolded numbers) neutralizing activity in the NOVAA-test is considered significant; 75–89% indeterminate; and below 75% uninterpretable. PRNT is considered positive when the titers is at least 10 (bolded numbers).

Among the 33 Polynesian subjects hospitalized for acute Dengue, the NOVAA-test was more specific, with only 1 falsely reactive sample and 3 in the gray area (88%). The PRNT was falsely reactive in 4 cases (12%) (Table 4).

**Table 4:** NOVAA-test and PRNT in 33 sera collected during acute clinical Dengue from hospitalized Polynesian children and adults (2015–2016).

ID	Age (y)	IgM Index	IgG Index	Neutralization NOVAA-Test %	PRNT titer
CHPF-01	7	positive	negative	28,45	<10
CHPF-02	60	positive	negative	0	<10
CHPF-03	14	positive	negative	9,59	<10
CHPF-04	23	positive	negative	0	<10
CHPF-05	42	positive	negative	9.59	<10
CHPF-06	50	positive	positive	49.68	<10
CHPF-07	13	positive	positive	**94.85**	**10**
CHPF-08	50	positive	positive	31.03	<10
CHPF-09	8	positive	positive	85.91	**10**
CHPF-10	53	positive	positive	18	<10
CHPF-11	55	positive	positive	57.33	<10
CHPF-12	68	positive	positive	0	<10
CHPF-13	67	positive	positive	14.22	<10
CHPF-14	35	positive	negative	22.41	<10
CHPF-15	6	positive	positive	12.5	<10
CHPF-17	7	positive	positive	76.83	<10
CHPF-19	7	positive	positive	68	**10**
CHPF-20	1	positive	positive	0	<10
CHPF-21	49	negative	positive	62.28	<10
CHPF-22	34	negative	positive	23.81	<10
CHPF-23	14	negative	positive	32.97	<10
CHPF-24	9	negative	positive	41.16	<10
CHPF-25	39	negative	positive	45.26	<10
CHPF-26	38	negative	positive	38.15	<10
CHPF-27	69	negative	positive	0	<10
CHPF-28	17	negative	positive	23.28	<10
CHPF-29	64	negative	positive	0	<10
CHPF-30	52	negative	positive	29.96	<10
CHPF-31	19	negative	positive	39.87	<10
CHPF-32	62	negative	positive	85.84	**10**
CHPF-33	52	negative	positive	7.97	<10
CHPF-34	29	negative	positive	0	<10
CHPF-35	66	negative	positive	0	<10

Positivity criteria: 90–100% (bolded numbers) neutralizing activity in the NOVAA-test is considered significant; 75–89% indeterminate; and below 75% uninterpretable. PRNT is considered positive when the titer is at least 10 (bolded numbers).

Among the 23 Dengue IgG antibody carriers negative by Dengue RT-PCR and negative for anti-Dengue IgM, specificity was 100% with both assays.

## Discussion

Use of pseudovirus technology to produce VLPs capable of a single infective cycle is yielding diagnostic tools that avoid the need to work in a high-level confinement laboratory [22]. The preliminary results obtained here with WNV/YFV17D VLP, in which the West Nile virus envelope is replaced with a yellow fever virus envelope, allow us to propose an alternative method for the detection of yellow fever neutralizing antibodies. Neutralizing antibodies induced by yellow fever vaccination shows a high degree of inter-individual variability [23]. The PRNT test detects a large panel of different epitopes from a living virus but is difficult to standardize and requires highly trained staff to estimate plaque reduction. It is also time-consuming and can only test a limited number of sera in a given run.

Comparison of the Asibi and 17D-204 genomes identified 67 nucleotide differences, 12 being located in the envelope E protein gene [17]. Interestingly, pseudovirus technology to produce VLP with the wild Type Asibi.strain envelope could be also of interest in order to better understand the immunogenicity and protection following the vaccination.

Standardization of the antigenic glycoproteins produced by the pseudovirus in these VLPs ensures a highly reproducible inoculum. The Vero cell line was chosen for its simple culture, robustness and, above all, its worldwide commercial availability, allowing the NOVAA-test to be standardized among its different users.

The different VLPs showed similar infectivities, as the inter- and intra-test repeatability shows that this test is adequate in terms of accuracy and repeatability. Production of a VLP stock is a simple and robust procedure. VLP production is subject to biological variability and its use for a same batch must be adapted according to the volume produced. In our experience, a VLP should be able to infect 20% of cells for comfortable reading. This implies the use of a variable volume of inoculum depending on the batch used.

The NOVAA-test is at least as sensitive as the PRNT test. All sera positive by PRNT collected at M12, whatever their titres, were highly reactive in the NOVAA-test, with more than 90% of neutralizing activity against the VLP pseudotype.

To determine the sensitivity of the NOVAA-test for samples with low PRNT titers, we tested 20 sera stored in our laboratory with known PRNT titers, including some with low titers. We also diluted 1:4 four sera with original PRNT neutralisation titers of 40, in order to approach the PRNT positivity cutoff of 10. Percentage neutralisation was always above 90% in the NOVAA-test (Fig 1).

Inhibition in the range 75–89% should be considered indeterminate pending further studies. There is extensive cross-reactivity between flaviviral infections [24]. In order to determine the specificity of the NOVAA-test, it was necessary to obtain panels of sera from patients free of all flaviviral infections and from patients with acute flavirus infections such as Dengue but unexposed to the yellow fever virus or vaccine. The NOVAA and PRNT tests were strictly negative in all the adults never exposed to flavivirus infection or vaccination. In contrast, specificity was limited during acute Dengue among patients from the Caribbean and Polynesia. Interestingly, reactivity was higher among Caribbean patients, as other unrecognized prior flaviviral infections could not be ruled out. Conversely, some time after acute Dengue infection, as confirmed by IgM and PCR negativity, the sera no longer cross-reacted in the NOVAA or PRNT test.

The NOVAA-test with its reporter gene allows standardized determination of a large number of sera by flow cytometry and is well suited to large epidemiological studies. The simplicity and high throughput of this test, the extremely small samples, and the availability of results within 3 days, make it an additional tool in the fight against yellow fever.
